# New Insights into Protein Kinase B/Akt Signaling: Role of Localized Akt Activation and Compartment-Specific Target Proteins for the Cellular Radiation Response

**DOI:** 10.3390/cancers10030078

**Published:** 2018-03-18

**Authors:** Klaudia Szymonowicz, Sebastian Oeck, Nathalie M. Malewicz, Verena Jendrossek

**Affiliations:** 1 Institute of Cell Biology (Cancer Research), University of Duisburg-Essen Medical School, 45122 Essen, Germany; klaudia.al-refae@uk-essen.de (K.S.); sebastian.oeck@uk-essen.de (S.O.); 2 Department of Therapeutic Radiology, Yale University School of Medicine, New Haven, CT 06520, USA; 3 Department of Anesthesiology, Yale University School of Medicine, New Haven, CT 06520, USA; nathalie.malewicz@yale.edu

**Keywords:** Protein Kinase B, Akt, PI3K, cellular radiation response, signaling network, Akt targets

## Abstract

Genetic alterations driving aberrant activation of the survival kinase Protein Kinase B (Akt) are observed with high frequency during malignant transformation and cancer progression. Oncogenic gene mutations coding for the upstream regulators or Akt, e.g., growth factor receptors, RAS and phosphatidylinositol-3-kinase (PI3K), or for one of the three Akt isoforms as well as loss of the tumor suppressor Phosphatase and Tensin Homolog on Chromosome Ten (PTEN) lead to constitutive activation of Akt. By activating Akt, these genetic alterations not only promote growth, proliferation and malignant behavior of cancer cells by phosphorylation of various downstream signaling molecules and signaling nodes but can also contribute to chemo- and radioresistance in many types of tumors. Here we review current knowledge on the mechanisms dictating Akt’s activation and target selection including the involvement of miRNAs and with focus on compartmentalization of the signaling network. Moreover, we discuss recent advances in the cross-talk with DNA damage response highlighting nuclear Akt target proteins with potential involvement in the regulation of DNA double strand break repair.

## 1. Introduction

Cancer has the highest incidence and causes the most mortality worldwide [1]. The phosphatidylinositol 3-kinase (PI3K)/Protein Kinase B (PKB, Akt) pathway is considered to be one of the most frequently altered pathways in human cancer with documented high relevance to tumor growth, metastatic spread, and resistance to anticancer treatment [2,3,4]. Many human solid tumors harbor genetic alterations driving aberrant activation of the survival kinase Akt such as, oncogenic activation of receptor tyrosine kinases, RAS or phosphatidylinositol-3-kinase (PI3K), loss of the tumor suppressor PTEN, or genomic amplification or gain-of-function mutations in one of the three known human Akt isoforms Akt1 (PKBα), Akt2 (PKBβ) and Akt3 (PKBγ) [5,6,7,8].

The three human Akt isoforms, Akt1, 2, 3, differ only in a few amino acids [9,10]. Akt1 was reported to be widely expressed in the whole body, Akt2 expression was markedly increased in insulin-dependent tissues and Akt3 was mostly observed in brain [11]. In vivo studies revealed tissue-related disabilities in animal experiments with Akt1^−/−^ mice being smaller and showing enhanced lethality [12].Instead Akt2-knockout resulted in a syndrome similar to diabetes [12], and Akt3 deficient mice were characterized by smaller brain size [13]. Double knockout of Akt1 and Akt2 was lethal after birth, whereas combined deletion of Akt1 and Akt3 even led to embryonic death [10,14,15]. Furthermore, combined targeted deletion of Akt1 and Akt2 in adult mice induced rapid mortality, whereas targeted deletion of Akt1 and Akt3 was tolerated [16]. These differences in the survival function of the three isoforms suggest that Akt isoforms may have different functions during development and adult life. These differences may be linked to tissue-specific differences in isoform expression or a distinct spectrum of target proteins. Of note is a recent analysis of copy number variations of the three Akt isoforms across multiple cancer types. This study revealed that amplification of Akt3 but not of Akt2 or Akt1 can be detected in diverse cancers, e.g., glioblastoma, melanoma, ovarian and breast cancer [17]. Since initial work has mostly been performed without specification of the isoform(s) or with Akt1, in this review the term “Akt” will be used synonymously with panAkt unless specifically mentioned otherwise.

**Figure 1:**
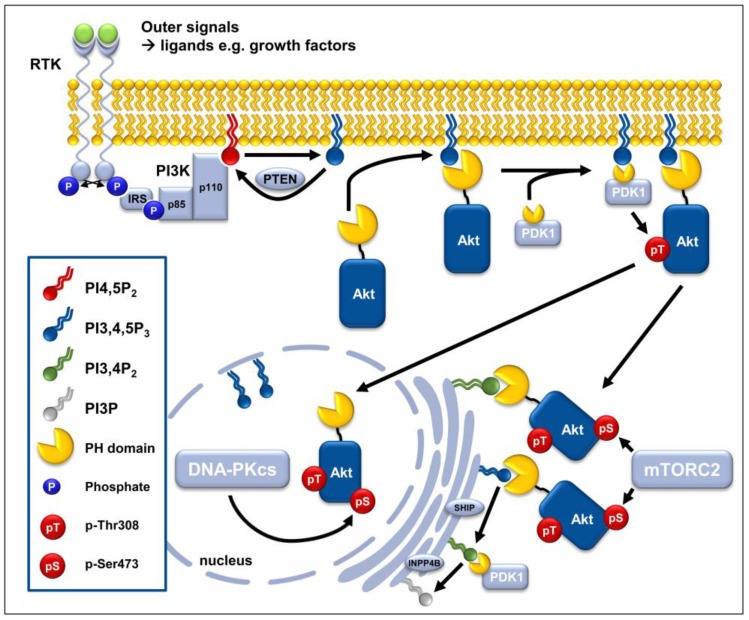
Schematic representation of compartmentalized Akt activation. PI(3,4,5)P_3_ production at the cell membrane depends on PI3K that is activated downstream of active RTKs and GPCRs. Subsequently, Akt is recruited to the cell membrane through binding of its PH-domain to synthesized PI(3,4,5)P_3_. Akt’s binding to PI(3,4,5)P_3_ allows phosphorylation at T308 by the PH-domain containing PDK1 and at S473 by mTORC2 or DNA-PKcs at distinct subcellular localizations. Akt can also be activated at endomembranes by binding to PI3,4P_2_ synthesized during a SHIP-catalyzed reaction from PI(3,4,5)P_3_. Instead, PTEN and INPP4B terminate Akt activation by limiting PI(3,4,5)P_3_ and PI(3,4)P_2_. (Inspired by [5,25]).

The serine and threonine kinase Akt plays an essential role in the regulation of various cellular functions, such as cell proliferation, cell growth, cell migration, and cell survival. Moreover, Akt is involved in the regulation of cell metabolism, protein synthesis, and immune cell function [18]. Therefore, it influences almost all aspects of cancer biology. Since active Akt promotes cell survival, particularly under stress conditions, including genotoxic stress, hyper-active Akt in malignant tumors has clinical relevance to the outcome of cancer therapy, e.g., by increasing resistance to chemo- and radiotherapy [5,19,20,21,22,23,24]. Here, we present current knowledge on the regulation of the multifaceted Akt signaling network including compartmentalized activation (Figure 1). 

Additionally, we discuss known effector proteins of Akt with potential impact on survival of irradiated cells and highlight nuclear Akt target proteins with a suggested role in the regulation of DNA double strand break repair.

## 2. Localized Regulation of Akt Activation and Activity

### 2.1. Phosphorylation and Activation of Akt

The main activation of Akt is done by PDK1 at cellular membranes initiated by growth factor-induced or insulin-induced activation of lipid kinases of the class I PI3K family. Further effectors for Akt phosphorylation are mTORC2 and DNA-PKcs with differing roles in the activation process. Additional phosphorylation sites of Akt, as well as various further sites for posttranslational modification have also been identified.

#### 2.1.1. PI3K-Dependent Phosphorylation of Akt at the Cytoplasmic Membrane

The PI3K family catalyzes the formation of the lipid second messenger phosphatidylinositol-3,4,5-trisphosphate (PI(3,4,5)P_3_) from phosphatidylinositol-4,5-diphosphate (PI(4,5)P_2_) [26] (see Figure 1). Herein, various extracellular stimuli initiate recruitment and activation of class I PI3Ks at receptor tyrosine kinases (RTKs) and G-protein coupled receptors (GPCRs). Subsequently PI(3,4,5)P_3_ recruits proteins harboring Pleckstrin Homology (PH)-domains such as Akt and phosphoinositide-dependent protein kinase 1 (PDK1) to the cell membrane. This brings both kinases in close vicinity and thereby promotes PDK1-dependent phosphorylation of Akt1 at T308 in the activation loop of the catalytic domain [27,28]. Thus, growth factor-dependent activation of Akt is strongly connected to the PI(3,4,5)P_3_ and PH-domain-dependent recruitment of Akt and its activating kinase to the cell membrane and the ability of PI3K to link activation of growth factor receptors to the formation of PI(3,4,5)P_3_ [27]. However, further work revealed that Akt can also be recruited via its PH-domain to sites of phosphatidylinositol-3,4-diphosphate (PI(3,4)P_2_) accumulation, presumably with preference at endomembranes [29,30]. PI(3,4)P_2_ can either be generated from PI(3,4,5)P_3_ by the action of the 5′-lipid phosphatase SHIP (SH-2-domain-containing inositol 5′-phosphatase) or be synthesized from phosphatidylinositol-4-phosphate (PI(4)P) by the action of Class II PI3Ks [25,27,31]. PH-domain-dependent relocalization of Akt to sites of PI(3,4,5)P_3_ or PI(3,4)P_2_ triggers conformational changes in Akt thereby allowing PDK1 to gain access to T308 [32,33].

Phosphorylation at T308 increases the activity of Akt1 about a hundredfold [34], but to gain a further ten-fold increase in kinase-activity and thus reach maximum activity, Akt has to be additionally phosphorylated at S473 in the hydrophobic motif by a member of the so-called PDK-2 family [35,36,37]. S473 has also been suggested to stabilize T308 phosphorylation [37,38]. Different kinases or even auto-phosphorylation are suspected to mediate S473 phosphorylation, presumably in a context-dependent manner. Amongst others, the phosphoinositide-dependent Mammalian Target of Rapamycin complex 2 (mTORC2), inhibitor of κB-kinase (IKK)-α, DNA-dependent protein kinase catalytic subunit (DNA-PKcs) and Integrin-linked kinase (ILK) have been proposed to act as so-called PDK2 and to phosphorylate Akt1 at S473 [24,35,39,40,41,42,43,44].

mTORC2 is considered to be the main activating kinase of Akt at S473. The following components of this multiprotein complex have been defined: Sin1, PRR5, Rictor, GßL, DEPTOR and mTOR [25,35,45]. The role of mTORC2 as so-called PDK2 phosphorylating Akt1 at S473 had been demonstrated using an in vitro kinase assay with the two mTORC2-components, mTOR and Rictor. These assays revealed that Rictor is essential for phosphorylating Akt at S473, whereas substitution by Raptor was not effective in inducing Akt phosphorylation, neither at S473 nor at T308 [35]. Ablating Sin1, another component of mTORC2, also strongly inhibited S473 phosphorylation and disrupted mTOR and Rictor-interactions; this suggests a key role of Sin1 in inducing activation of Akt at the hydrophobic S473 motif. In contrast, Sin1-ablation did not affect T308 phosphorylation, demonstrating that T308 activation can occur independently of prior phosphorylation at S473 [39,40]. Moreover, cells from Sin1−/− mice were also still able to phosphorylate the documented Akt target protein GSK3β [39]. Considering that Akt phosphorylation at T308 has the most pronounced effect on Akt activation, whereas phospho-S473 might stabilize or increase this initial Akt activation step, it is tempting to speculate that activation of Akt at T308 is sufficient to trigger GSK3β-activation at least to a certain extent. Alternatively, GSK3β could be a specific target of phospho-Akt-T308 that is independent from Akt’s-phosphorylation state at S473.

#### 2.1.2. mTORC2-Dependent Activation of AKT at Subcellular Membrane Compartments

It had been postulated that activity of PI3K is required for growth factor–induced and mTORC2-dependent activation of Akt. Herein, the localized production of PIPs seems to influence the activation of mTORC2, justifying its designation as “PDK2”. In this context, binding of PI(3,4,5)P_3_ to the PH-domain of the Sin1-component of mTORC2 was proposed to relieve the suppressive action of Sin1 on mTOR kinase activity and to trigger mTORC2 activation in response to growth factors [41,46,47]. In contrast, the work from Ebner and colleagues about the subcellular localization of mTORC2 revealed the constitutive presence of a growth factor- and PI3K-independent catalytically active subpopulation of mTORC2 at the plasma membrane and the mitochondria [48]. Instead the presence of a functional PI3K was only necessary for mTORC2-phosphorylation at early and late endosomes [48]. Moreover, though the PH-domain of Sin1 was required to target mTORC2 to the plasma membrane and to a subpopulation of endosomal vesicles, binding of Sin1 to cellular membranes occurred independently of PI(3,4,5)P_3_. This corroborates earlier in vitro findings that the PH-domains of Sin1 binds to 3- and 4-phosphorylated phosphoinositides without preference for PI3K products [48,49,50]. Importantly these findings uncovered several intracellular membrane compartments with mTORC2-activity being distinctly regulated by PI3K. The intracellular localization of mTORC2 therefore impacts the regulation of its activity towards Akt in the presence or absence of growth factor stimulation. The authors speculated that a constitutively active mTORC2 at the plasma membrane will facilitate full activation of Akt upon growth factor stimulation. The positive feed-back loop between Akt and mTORC2 is mediated by Akt-phosphorylation via Sin1. This may allow intracellular trafficking of active mTORC2 towards Akt and its specific substrates at distinct subcellular membrane compartments [46,48]. In this context endomembrane pools of PI(3,4,5)P_3_ and PI(3,4)P_2_ have been described, that may facilitate intracellular activation of Akt [25,51,52]. 

Further observations support a context-dependent regulation of Akt activation: for example JEG-3 placental choriocarcinoma cells responded to tunicamycin-induced endoplasmic reticulum (ER) stress by an inverse regulation of Akt phosphorylation: here phosphorylation at S473 was enhanced whereas phosphorylation of T308 was decreased [53]. Similarly, an alternative mechanism of Akt activation by a substrate-docking motif, the so-called “PIF-pocket mechanism” is initiated by mTORC2-dependent phosphorylation of Akt at S473, whereas recruitment of PDK1 to phosphorylate T308 occurs as a secondary step [54].

#### 2.1.3. DNA-PKcs-Dependent Phosphorylation of Akt in the Nuclear Compartment

Further reports identified DNA-PKcs as an upstream kinase for Akt1 phosphorylating S473 under certain conditions including DNA damage [23,24,36,55,56,57]. Indeed, the phosphorylation hierarchy between DNA-PKcs and Akt upon DNA damage was controversial for a long time. Using immunoprecipitation, Toulany and colleagues described a physical connection between the two kinases pointing towards the role of Akt in the regulation of DNA double strand break (DSB) repair by non-homologous end joining (NHEJ) [42]. Accordingly, inhibition of Akt1 expression resulted in decreased phosphorylation of DNA-PKcs at S2056 and T2609. Furthermore, pharmacological inhibition of Akt1 not only reduced cell survival upon radiation-induced DNA damage, but also slowed the repair of DNA DSB in DNA-PKcs-proficient cells. The authors concluded that Akt1 acts in a signaling network upstream of DNA-PKcs. In contrast, treatment with the DNA-PKcs inhibitor NU7026 reduced S473 phosphorylation of Akt upon irradiation or doxorubicin treatment, whereas DNA-PKcs inhibition did not affect Akt S473 phosphorylation in human umbilical vein endothelial cells treated with insulin [24]. DNA-PKcs did also phosphorylate Akt1 at S473 upon treatment with cis-platin or ionizing radiation, but not with insulin [24,58,59]. A proof for DNA-PKcs mediated phosphorylation of the hydrophobic Akt motif S473 was obtained using purified peptides containing the S473 motif [36,55]. Consistent with these findings, our own investigations revealed that DNA-PKcs does in fact phosphorylate Akt1 directly at S473 in the presence of damaged DNA but not vice-versa [23]. Combined, these reports highlight an interaction of DNA-PKcs and Akt1 in cells experiencing DNA damage to facilitate DNA-repair [23,24,42,55,60].

#### 2.1.4. Additional Phosphorylation Sites of Akt

Akt’s activity and function are connected to a balanced system of phosphorylation and dephosphorylation. For many years S473 and T308 phosphorylation sites were considered as rate limiting, necessary, and sufficient for Akt1 activation [37]. Of note, the related isoforms activation occurs at the corresponding residues T309/S474 in Akt2 and T305/S472 in Akt3 [25].

Meanwhile at least two additional phosphorylation sites—S477 and T479 [61]—as well as various further sites for posttranslational modification have been identified [61,62,63,64,65]. For instance, mTORC2-dependent phosphorylation at T450 in the turn-motif seems to be required for proper folding of Akt [64,65]. Furthermore, S477 and T479 in the regulatory domain impact Akt’s activation state. S477A and T479A phosphorylation-ablating mutants not only affected Akt1 kinase activity towards GSK3β, but also strongly impaired phosphorylation at T308 and S473 [61]. Similar observations were made for the respective phosphorylation sites in the Akt2 and Akt3 isoforms. These findings suggest, that both newly discovered phosphorylation sites are essential to achieve full Akt activation. 

Of note, Cdk2/Cyclin A2 exclusively phosphorylated Akt at S477 and T479 in a cell cycle dependent manner. In contrast, mTORC2 and DNA-PKcs may not only phosphorylate Akt at S473 but also at S477 and T479 under conditions of growth factor stimulation or DNA damage, respectively [61]. Moreover, phosphorylation of Akt by the protein kinase CK2 at S129 was described to enhance Akt’s catalytic activity [63] whereas phosphorylation of T312 by glycogen synthase kinase α (GSK3α) attenuated its activity [66].

##### Perspective

The above findings imply that in the presence of insulin or growth factors mTORC2 functions as the main PDK2 to phosphorylate Akt at S473. In contrast, DNA-PKcs is responsible for the phosphorylation of Akt-S473 in response to DNA damage, presumably in the nucleus or at the nuclear-cytoplasmic interface. It is intriguing that PI(3,4,5)P_3_ is also enriched at endomembranes such as the early endosome and the nuclear envelope [51,67]. This could be critical for localized activation of Akt and thus specificity in the activation of Akt substrates in specific subcellular compartments as well as the context-dependent timing and duration of specific Akt responses. For example, endosomal Akt has been linked to the trafficking of growth factor receptor complexes and thus the control of spatiotemporal regulation of their activity [68]. Finally, DNA-PKcs has been shown to activate Akt as so-called PDK2 in the nucleus upon DNA damage. However, there are multiple reports about nuclear PI3K/phosphoinositide signaling [69,70,71]. Thus, it is tempting to speculate that nuclear PI3Ks and nuclear PIP-pools, or even nuclear phosphoinositide-phosphates organized within membrane bilayers of the nuclear envelope protruding into the nuclear matrix as part of invaginations, may alternatively allow for the localized activation of Akt and subsequent phosphorylation of nuclear Akt substrates involved for example in DNA replication and DNA repair [69,70,71]. However, the function of these molecules and their effector pathways is still poorly understood and needs further investigation [72]. The additional phosphorylation sites might participate in the fine-tuning of Akt activation as well as the strength and duration of Akt’s kinase activity towards its substrates. Posttranslational Akt modifications may also influence downstream cellular events by modulating Akt’s subcellular localization. For example, its ability to translocate into the nucleus and thereby gaining access to nuclear target proteins [73,74].

### 2.2. Termination of Akt Activity by Dephosphorylation

Two families of phosphatases participate in the termination of Akt activity: (i) The 3′-lipid phosphatase PTEN as well as the 4′-lipid phosphatase inositol polyphosphate-4-phosphatase (INPP4B) remove the activating lipid messengers PI(3,4,5)P_3_ and PI(3,4)P_2_ resulting in reduced membrane recruitment and activation of Akt [75]. (ii) Various protein phosphatases such as the pleckstrin homology domain leucine-rich repeat protein phosphatases (PHLPP1/2) and protein phosphatase 2A (PP2A) inhibit Akt by dephosphorylation of S473 or T308, respectively [25].

#### 2.2.1. Lipid Phosphatases PTEN and INPP4B

Based on its ability to convert PI(3,4,5)P_3_ into PI(4,5)P_2_ the PI3K antagonist PTEN counteracts membrane recruitment of PH-domain containing proteins such as Akt and PDK1, and thereby precludes PDK1-dependent phosphorylation of Akt at T308 in a cell-type and context-dependent manner [76]. Cancer cells carrying loss of function mutations in PTEN lack this regulatory function and are therefore characterized by aberrant and constitutive T308-phosphorylation and thus activation of Akt, and accelerated proliferation [77,78,79]. Surprisingly, in rhabdomyosarcoma cell lines are an inverse correlation between PTEN levels and Akt1 phosphorylation at S473 but not T308 has been observed [80]. The levels of PTEN or of the active Akt1-mutant E17K did also not correlate with higher Akt-T308 phosphorylation in cells from patients suffering from acute myeloid leukemia [81]. These findings suggest that in some cells PTEN might also control Akt phosphorylation at S473, presumably via some indirect effects on mTORC2 signaling: For example a PI(3,4,5)P_3_-dependent recruitment of the PH-domain containing mTORC2-protein Sin1 has been proposed to facilitate S473-phosphorylation [47,48]. Moreover, PTEN-depletion has been associated with decreased phosphorylation of Rictor at T1135 resulting in improved formation and activation of mTORC2 and enhanced downstream activation Akt in glioblastoma multiforme [82].

The action of the 4′ lipid phosphatases INPP4B hydrolyzes PI(3,4)P_2_ to PI(3,4,5)P_3_ and can also abrogate membrane recruitment and activation of Akt [83,84]. Interestingly, PI(3,4,5)P_3_ can instead activate serum and glucocorticoid-dependent kinase 3 (SGK3), presumably in a context-specific manner, but the importance of this process must be further explored [75]. Since PTEN and INPP4B can restrain oncogenic activation of Akt, it is not surprising that loss of PTEN or INPP4B expression or function promotes tumor growth in murine cancer models through enhanced Akt isoform-specific signaling [75].

#### 2.2.2. Protein Phosphatases PHLPP1/2, PP2A

Together with the upstream activating protein kinases PDK1, mTORC2, DNA-PKcs and ILK specific phosphatases create a direct Akt activation/deactivation cycle. Inactivating dephosphorylation of S473, S474 and S472 in the hydrophobic motifs of Akt1, Akt2 and Akt3 respectively, is ensured by PHLPP1/2 in an isoform-specific manner: While PHLPP1 dephosphorylates Akt isoforms 2 and 3, PHLPP2 is responsible for dephosphorylation of the Akt isoforms 1 and 3 [85,86]. Interestingly, PHLPP1/2 dephosphorylates Akt only at its hydrophobic motif [86,87]. PHLPP1 has been described as a tumor suppressor in prostate cancer [88] and there are preliminary findings that aberrant expression of PHLPP1/2 may be associated with poor prognosis in several cancers [89,90,91]. Protein phosphatase 2A (PP2A) constitutes another Akt deactivating phosphatase that preferentially mediates dephosphorylation of Akt at T308 [92,93,94]. PP2A was reported as a selective regulator for Akt1 phosphorylation at T308 [94]. Consequently, overexpression of a B55α subunit of PP2A strongly correlated with pT308 inhibition and decreased phosphorylation of Akt targets as well as cell proliferation [94]. Moreover, Akt can be directly dephosphorylated at S473 or both, S473 and T308 by protein phosphatase 1A (PP1A) [95,96].

##### Perspective

The genetic make-up of specific cancer cells and cell lines impacts Akt regulation by protein kinases, protein phosphatases and lipid phosphatases [36,55,56,57,97]. Interestingly, the duration of Akt activation and Akt-mediated phosphorylation of target proteins is restricted in a spatiotemporal manner. For example, Akt signaling in the cytosol is more rapid and more transient compared with the cell membrane and the nucleus, suggesting the presence of differentially regulated kinase and phosphatase activity in the different compartments [32]. It appears that spatiotemporal intracellular segregation of active kinases and phosphatases may participate in the control of Akt phosphorylation and its localized access to its targets.

### 2.3. Further Factors Regulating Akt Activation

In addition to phosphorylation and activation as well as termination of Akt activity by dephosphorylation, further regulation can be performed via factors like 5′lipid phosphatase, PH-domain containing proteins, AIM2 and HSP90. Posttranslational modification via ubiquitin-modifying enzymes of Akt could play a more refined regulatory role. Another novel aspect in the regulation of the PI3K/Akt pathway is the involvement of miRNAs.

#### 2.3.1. 5′-Lipid Phosphatases

As outlined above 3′-lipid phosphatase PTEN as well as INPP4B terminate Akt activation by limiting PI(3,4,5)P_3_ and PI(3,4)P_2_. Instead, 5′lipid phosphatases such as SHIP or INPP5D trigger the formation of PI(3,4)P_2_, which is still able to induce re-localization of Akt to cellular membranes via engagement of the PH-domain (Figure 1) [25,52].

#### 2.3.2. PH-Domain Containing Proteins

Furthermore, PH-domain-only proteins such as the p53 target protein PHLDA3 can compete with the PH-domain of Akt for binding to membrane lipids, thereby inhibiting Akt’s membrane translocation and thus activating phosphorylation; vice-versa deficiency of endogenous PHLDA3 can result in increased Akt activity and apoptosis resistance [98]. Interestingly, PHLDA3 and INPP5D constitute two hypoxia-induced pro-apoptotic p53 target genes that reduce Akt phosphorylation and promote apoptosis in p53-proficient cancer cells under hypoxic conditions [99].

#### 2.3.3. AIM2 and HSP90

Additionally, Akt activity can be controlled by factors limiting activation of its upstream regulatory kinases mTORC2 [100] or DNA-PKcs such as the innate immune sensor absent in melanoma 2 (AIM2) [97]. Moreover, chaperone proteins such as heat shock protein 90 (HSP90) can interact with phosphorylated Akt to maintain the kinase in a stable activation state [92]. While, HSP90 is up-regulated in many diseases including cancer, treatment with HSP90 inhibitors evoked dissociation of HSP90 from phosphorylated Akt1 and subsequent degradation, highlighting a new potential therapeutic target for the treatment of cancer with up-regulated Akt activity [92,101].

#### 2.3.4. Ubiquitin-Modifying Enzymes

Ubiquitin-modifying enzymes seem to play an important modulatory role in posttranslational modification of Akt at various Lysine residues possibly accounting for further fine-tuning and spatiotemporal regulation of Akt activity and potentially substrate specificity [21]. It has been suggested that two E3 ligases, CHIP (C-terminus of HSC70-Interacting Protein) and MULAN (mitochondrial ubiquitin ligase activator of NF-κB) may terminate Akt activity by triggering its cytosolic K48-directed polyubiquitination and subsequent degradation [102,103]. However, this assumption remains controversial as others have revealed a correlation between the overexpression of CHIP and elevated Akt activity [90]. Except from CHIP and MULAN, the related K48-linked ubiquitination system involves BRCA1 (breast cancer susceptibility gene 1) and TTC3 (tetratricopeptide repeat domain 3). BRCA1 can bind to phosphorylated Akt1 and promote its K48-linked ubiquitination mainly in the nucleus thereby inducing proteasomal degradation of Akt1 [73,74,104,105]. In contrast K63-linked ubiquitination leads to activation of Akt1. It involves TRAF6, Skp2 (SKP1 interacting partner 2) and NEDD4-1 enhanced nuclear translocation of Akt1 after its prior phosphorylation (pT308/pS473) by PDK1 and mTORC2 [73]. While TRAF6 promoted the activation and ubiquitination of Akt1 downstream of IGF-1 signaling [106], and the E3 ligase-Skp2 mediated signals received from EGFRs [104], NEDD4-1-mediated ubiquitination of TRAF3 was shown to promote CD40-dependent Akt1 activation in an indirect manner [107].

#### 2.3.5. miRNA

miRNAs are defined as single-stranded noncoding RNA fragments composed of approximately 22 nucleotides and generally regulate protein expression through gene silencing. In cancer cells miRNAs can facilitate or suppress tumor growth, angiogenesis and invasion. Interestingly, miRNAs alter expression and function of signaling proteins of the PI3K/Akt pathway and radiosensitivity [108,109]. Up-regulation of specific miRNAs, e.g., miR-20a, miR-21, miR-22, miR-95, miR-106b, miR-205, miR-519a, has been associated with up-regulated expression or activity of the PI3K and increased proliferation, poor prognosis or even radioresistance in various tumors, e.g., hepatocellular carcinoma, colorectal cancer, nasopharyngeal carcinoma, chronic lymphocytic leukemia, or lung cancer, mostly by down-regulating PTEN [110]. In contrast, down-regulation of specific miRNAs, that limit PI3K/Akt pathway activation e.g., miR-31, miR-126, miR-203, miR-302, have similarly been associated with up-regulated activity of Akt, increased tumor cell proliferation and radioresistance in undifferentiated thyroid carcinoma, lung adenocarcinoma, nasopharyngeal carcinoma and malignant glioma, or breast cancer, respectively [111,112,113,114,115]. Reports about the miRNA effects on Akt regulation are summarized in Table 1.

##### Perspective

Ubiquitination and miRNA regulation of Akt activity constitute two new intriguing aspects in the regulation of Akt activity. Herein, particularly the suggested interplay between Akt phosphorylation and K63-linked ubiquitination in the regulation of nuclear translocation might play an important role in the cellular radiation response as it could facilitate the access of Akt to nuclear target proteins. Importantly, phosphorylated Akt1 and not panAkt1 mainly underwent NEDD4-1-dependent ubiquitination upon stimulation of MCF7 cells with insulin or insulin growth factor; moreover, NEDD4-1-dependent ubiquitination of the phospho-Akt1 mutant E17K with enhanced affinity to the cellular membrane was enhanced in all cellular compartments compared to wild type phospho-Akt1, and associated with enhanced nuclear trafficking towards nuclear or perinuclear compartments [74]. However, since a phosphorylation-defective Akt1 mutant (T308A/S473A) still entered the nucleus, phosphorylation of Akt1 may be dispensable for its nuclear translocation and only accelerate the translocation process [121,122]. Yet the role of Akt phosphorylation and subsequent K63-linked ubiquitination for its subcellular localization requires further definition.

## 3. Subcellular Network of Akt Target Proteins

### 3.1. General Aspects of Target Protein Selection

Akt phosphorylates its substrates primarily at a specific Akt consensus phosphorylation motif RXRXXS/T that is followed at preference by a large hydrophobic residue [25,37]. So far, more than 100 putative Akt target proteins have been described [123]. Of note, some of the Akt target proteins such as Mdm2 get activated upon Akt-mediated phosphorylation and thus function as Akt effector proteins, whereas other factors become inactivated, e.g., GSK3β, FOXO3a [124,125].

#### 3.1.1. Importance of the Akt Isoform for Target Protein Selection

So far only a few studies have investigated potential differences in the target proteins of the three Akt isoforms. For instance, the anti-apoptotic and EGF-dependent mitogenic effects have been mostly attributed to Akt1 and Akt3, whereas Akt2 seems to be mainly involved in the regulation of cell migration [10,126]. Interestingly, in a recent study, the Akt1 isoform was unable to induce PDGF-dependent high-grade glioma in a genetically engineered tumor model, whereas Akt2 and Akt3 isoforms strongly promoted high-grade glioma progression [8]. The authors could link differences in polarity and hydropathy values of the pleckstrin homology domain and the regulatory domain between the Akt isoforms to the tumor-promoting effects of Akt2 and Akt3. Furthermore, tumors driven by distinct Akt isoforms were characterized by pronounced differences in gene and protein expression: Of note, a particularly high expression of DNA repair protein-encoding genes like polymerase delta 1 (PolD), DNA polymerase epsilon (PolE), Exonuclease 1 (EXO1), Proliferating cell nuclear antigen (PCNA), and Flap endonuclease 1 (FEN1) were observed in Akt3-derived high-grade glioma and might explain their increased resistance to radiotherapy and chemotherapy [8]. A complementary study focused on the influence of Akt isoforms radiosensitivity and DNA repair in colon cancer cells. The authors revealed that knockout of Akt1, Akt2 or both increased radiation sensitivity and that Akt1/Akt2 double knockout impaired the rejoining of radiation-induced DNA double strand breaks (DSB) via DNA-PKcs-dependent cNHEJ [127].

#### 3.1.2. Importance of Akt’s Phosphorylation State for Target Protein Selection

Interestingly, the efficiency of Akt in target protein phosphorylation seems to depend on the phosphorylation state of Akt and is dictated by the cellu

lar context as described above: For example, in insulin treated or serum starved Sin1-/- murine embryonic fibroblasts with deficiency in mTORC2 function phosphorylation of GSK3β and TSC2 did not depend on S473 phosphorylation of Akt, whereas phospho-S473 was necessary for Akt-dependent phosphorylation of FOXO1/3a [39]. In contrast, in an in vitro system T309 phosphorylation of Akt2 was required to phosphorylate peptides of the paradigmatic Akt substrates FOXO3a and GSK3β whereas additional S474 phosphorylation was only required to phosphorylate full-length GSK3β but not full-length FOXO3a [128]. Phosphorylation of at least two of the three further known Akt target proteins Proline-rich AKT1 substrate 1 (PRAS40), tuberous sclerosis 2 (TSC2) and TBC1 Domain Family Member 4 (TBC1D4) in non-small cell lung cancer cell lines also required Akt1-phosphorylation at T308 for their activation [129]. These findings suggest that phosphorylation at T308/T309 might be more critical for the kinase activity and subsequent substrate activation than phosphorylation at S473/S474 [130].

Nevertheless, this does not rule out that phosphorylation at S473/S474 may be required for maximal kinase activity and further increase in the extent of target phosphorylation as suggested by others [37,131]. Moreover, this does not preclude that phosphorylation of specific Akt target proteins may require additional S473 phosphorylation, as shown for inactivating phosphorylation of FOXO1/3a transcription factors [39]. In line with these findings, differences in the requirement of S473-phosphorylation for Akt-mediated regulation of glucose uptake via glucose transporters have been reported: Akt activation via insulin promoted glucose uptake into fat and muscle cells via the glucose transporter Glut4 to maintain glucose homeostasis and this requires only phosphorylation of Akt’s kinase domain at T308 via PDK1. Instead, enhanced glucose uptake via Glut1 is necessary for an anabolic metabolism during tissue growth and repair downstream of growth factor-induced Akt activation. Additionally, mTORC2-dependent phosphorylation of S473 in the hydrophobic motif domain is required. However, S473 phosphorylation seems to be more important for the phosphorylation of specific targets under specific conditions: for example, phosphorylation of GSK3β and HDM2 was enhanced under conditions of tunicamycin-induced ER stress in comparison to untreated cells in the presence of reduced phosphorylation of T308 and enhanced S473 phosphorylation [53]. 

#### 3.1.3. Importance of Subcellular Localization for Target Protein Selection

Though it is well established that Akt is activated by PDK1 and mTORC2 upon PI3K-dependent recruitment to the cytoplasmic membrane, there is increasing evidence that Akt can also be activated in distinct subcellular compartments. This may be crucial for phosphorylation of substrates with specific or distinct cellular localization and function. Phosphorylation of cytosolic target proteins, e.g., GSK3β, Mdm2, or p21 mostly stimulates proliferation and survival. But Akt can also accumulate in mitochondria, for example in response to insulin-like growth factor-1 (IGF-1)-stimulation and phosphorylate mitochondria-localized GSK3β [132]. In the context of tumor hypoxia, mitochondria-localized Akt was shown to phosphorylate pyruvate dehydrogenase kinase 1 on Thr346 thereby inhibiting formation of the pyruvate dehydrogenase [133].

Finally, an increasing number of publications reveal the ability of Akt to localize to the nuclear compartment making an involvement in regulatory processes in the nucleus and DNA repair highly likely. Furthermore, nuclear PI3Ks and nuclear phosphoinositide signaling might also allow for the localized activation of Akt and subsequent phosphorylation of nuclear Akt substrates involved for example in DNA replication and DNA repair but the function of these molecules and their effector pathways is still poorly understood and needs further investigation [51,69,70,71,72,134,135].

##### Perspective

We assume that substrate recognition and phosphorylation of Akt target proteins is based on a cell type- and context-dependent manner. In the following paragraphs, we will highlight current knowledge on Akt substrates with central functions as central nodes in cellular signaling networks and thus potential indirect roles in the cellular radiation response, as well as direct Akt substrates with suspected roles in the DNA damage response, including cell cycle regulation, cell death and DNA repair. The Akt substrates described below as well as further proteins with reported function as Akt target proteins are listed in Table 2.

### 3.2. Akt Substrates with Functions as Key Regulators of Cell Signaling Networks

Akt regulates various cellular functions by phosphorylating a wide variety of substrates presumably in a cell type- and context-dependent manner. In the following paragraphs we describe important Akt substrates and their role in cell function considering their subcellular localization.

#### 3.2.1. Glycogen Synthase Kinase 3, GSK3

The Akt target protein GSK3 has initially been described as a key enzyme regulating glycogen metabolism [175]. But nowadays the two GSK3 isoforms, GSK3α and GSK3β (glycogen synthase kinase 3 α/β), are known to exert multiple redundant as well as some isoform-specific functions in the regulation of cell metabolism, particularly glucose metabolism, cell cycle progression, cell division, cell survival, protein synthesis and microtubule function [125,176,177,178,179]. In addition to its role in the PI3K/Akt pathway GSK3 also functions in other pathways such as the Wnt/β-catenin pathway, presumably in a PI3K/Akt-independent manner [25,176]. GSK3 is a Ser/Thr kinase that is active in its non-phosphorylated form, and thus negatively regulates the activity or the stability of most of its various target proteins in the absence of growth factors, insulin and nutrients. Instead, growth factor-induced or insulin-induced activation of Akt promotes inactivating phosphorylation of GSK3α and GSK3β at S21 and Ser9, respectively, thereby abrogating their action as kinases [175]. Growth factor-, insulin- or oncogene-induced Akt activation will thus positively regulate the activity or function of the GSK3 substrates by switching-off the inhibitory or degradation-promoting action of GSK3 [25], but kinases other than Akt can also phosphorylate the same Ser-residues that are targeted by Akt to switch off GSK3 activity in a context-dependent manner [25,176].

For example, active GSK3 regulates cell metabolism and cell growth by phosphorylation and inhibition of metabolic enzymes such as glycogen synthase or eukaryotic initiation factor 2B (eIF2b), respectively. As a consequence, GSK3β-mediated inactivation of glycogen synthase will result in reduced glycogen synthesis and enhanced glycolytic rate [178], and inactivation of eIF2b will reduce protein synthesis [176]. But GSK3-dependent phosphorylation of other target proteins marks these substrates for ubiquitination and subsequent proteasomal degradation, for example proteins involved in the regulation of cell survival and proliferation such as the anti-apoptotic member of the Bcl-2 family Mcl-1, the cell cycle regulator Cyclin D1, or the transcription factor c-myc. Consequently, inhibitory phosphorylation of GSK3β by active Akt leads to increased stability and accumulation of these proteins resulting in apoptosis resistance, cell cycle progression and enhanced proliferation respectively [125,179,180,181,182]. Interestingly, Shimura and colleagues proposed that fractionated irradiation of HeLa or HepG2 cells evokes an Akt1-dependent DNA damage response-loop: Akt-dependent inhibition of GSK3β led to nuclear accumulation of Cyclin D1 and Cyclin D1-triggered S phase progression of cells with an enhanced amount of DNA DSBs that in turn trigger activation of DNA-PKcs, the key enzyme of cNHEJ, and finally DNA-PKcs-dependent Akt1 activation [180]. Instead, S-phase progression caused by overexpression Cyclin D1 had an inhibitory effect on DNA repair via homologous recombination repair (HRR) by recruiting Rad51, an essential protein involved in HRR [183]. Altogether these findings indicate that the Akt/GSK3 axis seems to play a yet unrecognized indirect role in the DNA damage response and DNA repair.

#### 3.2.2. FOXO Transcription Factors

Transcription factors of the Forkhead BOX O family (FOXO)—FOXO1, 3, 4 and 6, constitute further highly conserved targets of Akt [151,184]. They regulate the expression of multiple target genes involved in diverse cellular processes such as cell cycle progression, cell proliferation, catabolic metabolism, stress resistance, autophagy and apoptosis in a cell type and context-dependent manner [185,186,187,188]. Active Akt mostly counteracts FOXO function by phosphorylating the three conserved residues of FOXO1 (T24, S256, S319), FOXO3a (T32, S253, S315) and FOXO4 (T32, S197, S262). Herein the phosphorylation of a serine close to the nuclear localization sequence (S253 in FOXO3a) seems to be particularly important for the regulation of their subcellular localization: S253-phosphorylation masks the nuclear localization sequence and generates recognition sites for 14-3-3 export proteins thereby promoting FOXO’s nuclear exclusion. Moreover phosphorylation primes FOXOs polyubiquitination and subsequent proteasomal degradation [25,151,186]. Consequently, inhibition of Akt, for example by treatment with S-equol, a secondary metabolite of the natural anticancer isoflavone daidzein, prevents the degradation of FOXO3a and decreased prostate tumor growth [189]. However, controversial data has been obtained with the less specific inhibitor of the upstream kinase PI3K, LY294002 in other cell types suggesting cell type-dependent or Akt-independent mechanisms [151,190]. Since Mdm2 can promote ubiquitination and degradation of FOXO3a, Akt may also indirectly affect the function of the FOXOs via the regulation of Mdm2 [185]. Comparably to GSK3β-phosphorylation other kinases can participate in the phosphorylation of FOXOs. Moreover, the function of FOXO transcription factors is regulated by posttranslational acetylation and ubiquitination [186].

#### 3.2.3. TSC2, mTORC1, and PRAS40

The mechanistic target of rapamycin (mTOR), formerly “mammalian target of rapamycin”, is a central regulator of fundamental cell processes that coordinates growth and metabolism with growth factor signaling and nutrient provision downstream of PI3K/Akt signaling [191]. The Ser/Thr kinase mTOR constitutes the catalytic subunit of two multiprotein complexes, mTOR complex 1 (mTORC1) and 2 (mTORC2). mTORC1 is composed of the core components mTOR, Raptor (regulatory protein associated with mTOR), and mLST8 (mammalian lethal with Sec13 protein 8, also known as GßL) and contains also the inhibitory subunits PRAS40 (proline-rich Akt substrate of 40 kDa) [192,166,193] and DEPTOR (DEP domain containing mTOR interacting protein) [194]. mTORC2 shares the core components mTOR and mLST8 with mTORC1, but contains the rapamycin-insensitive companion of mTOR (Rictor) instead of the rapamycin-sensitive Raptor [195,196]. Further components of mTORC2 are DEPTOR [194] as well as the regulatory subunits Sin1 [39,197] and Protor1/2 [191]. The growth-promoting effects of Akt are primarily exerted via the signaling network initiated by mTORC1 [198]. Herein, mTORC1 promotes anabolic metabolic programs to ensure increased production of proteins, lipids and nucleotides needed for cell growth and division; at the same time mTORC1 senses and controls the balance between anabolic and katabolic processes including autophagy and the ubiquitin-proteasome system in dependence of intracellular availability of nutrients and energy as well as the respective environmental conditions. mTORC1 executes these functions with the help of two classes of Ras-related small G proteins, the Rag and Rheb GTPases as well as active Akt [191,195,199,200,201]. Instead mTORC2 controls growth and survival by phosphorylating kinases of the ACG family, particularly Akt, downstream of growth factor and insulin-mediated activation of PI3K as described in more detail above [35,191]. Thus, while Akt is a substrate of mTORC2, it acts upstream of mTORC1.

It had been suggested that active Akt directly phosphorylates mTOR at S2448 downstream of insulin treatment. However, these results remain controversial as this mTOR-residue is also a target of the kinase p70S6K that acts downstream of mTORC1 [25,202]. It is therefore more likely that mTORC1 is not a direct substrate of Akt. Instead there is accumulated evidence that active Akt inhibits the inhibitory action of the tuberous sclerosis complex (TSC) towards Rheb, a Ras-related GTPase that in its active GTP-bound form functions as an essential activator of mTORC1. The TSC complex is composed of tuberous sclerosis complex 1 and 2 (TSC1, hamartin; TSC2, tuberin) as well as Tre2-Bub2-Cdc16-1 domain family member 7 (TBC1D7) [203]. Active TSC triggers the formation of inactive Rheb-GDP that is unable to stimulate mTORC1. Thus, active TSC exerts inhibitory function towards mTORC1 and inhibits cell growth and protein synthesis [25,204,205]. Akt-dependent phosphorylation of TSC2 relieves its inhibitory GTPase activity towards Rheb, restores Rheb-GTP and activates mTORC1 [191,199,204,206]. However, activation occurs only in the presence of amino acids as they are necessary to load and activate the Rag GTPase complex at the lysosomal surface and thereby recruit mTORC1 to sites of active Rheb [193].

Akt also phosphorylates the inhibitory subunit PRAS40 at T246 thereby relieving its inhibitory action on Raptor. Raptor facilitating substrate recruitment allows for mTORC1 to phosphorylate its downstream targets p70S6K and 4E-BP1, resulting in enhanced protein synthesis and cell growth [191,193,207,208]. mTORC1 also enhances de novo lipid synthesis through the sterol responsive element binding protein (SREBP) transcription factor and of purine and pyrimidine nucleotides required for DNA replication and ribosome biosynthesis during cell growth and proliferation [191,209,210,211]. In addition to the regulatory action of mTORC1 on nucleotide provision its ability to respond to environmental cues is particularly interesting with respect to our understanding of how the Akt signaling network impacts the cellular radiation response. Environmental stress conditions that impair cell growth such as low levels of nutrients, energy (ATP), or oxygen (hypoxia), or DNA damage result in the inhibition of mTORC1 through activation of its negative regulator AMPK, or the induction of regulated in DNA damage and development 1 (RHEDD1) or via the p53 target genes AMPK regulatory subunit (AMPKβ), PTEN and/or TSC2, thereby triggering TSC activation [191,212]. Thus, the Akt/mTORC1 axis might also play an indirect role in the DNA damage response that needs to be further explored.

#### 3.2.4. Mdm2

Another target protein of Akt that is important to regulation of various cellular functions including the cellular radiation response is the oncogenic ubiquitin ligase Mdm2, the master regulator of the tumor suppressor p53. Akt-mediated phosphorylation of Mdm2 at S166 and S186 promotes nuclear translocation of Mdm2 thereby triggering ubiquitination, nuclear exclusion and cytoplasmic degradation of p53. Since p53 is crucial for the regulation of the DNA damage response, cell cycle arrest, cell metabolism, cellular senescence and cell death, Mdm2-induced p53-degradation will abrogate p53-induced effects on these functions and thus impact the cellular radiation response indirectly [124,213,214].

### 3.3. Direct Effector Proteins of Akt with Roles in Cell Cycle Regulation and Cell Death 

DNA double strand break (DSB) are the most lethal lesions induced by ionizing radiation [215]. Generally, DSB activate the kinases Ataxia telangiectasia and Rad3-related protein (ATR), Ataxia Telangiectasia Mutated (ATM) and DNA-PKcs and initiate cell cycle arrest and DNA repair or cell death/apoptosis via specific downstream signaling cascades [216,217,218]. Several direct target proteins of active Akt have been identified e.g., the cell cycle regulators p21Cip1/Waf1/Sdi1 and p27Kip1 as well as the pro-apoptotic Bcl-2 family members Bad (Bcl-2-associated agonist of cell death) and Bax (Bcl-2 associated X protein). These are involved in the effects of Akt on cell cycle progression and cell survival and may therefore impact the cellular radiation response [5,20,21,22]. In both studies Akt-dependent phosphorylation impacted subcellular localization of its substrates [219].

**Figure 2:**
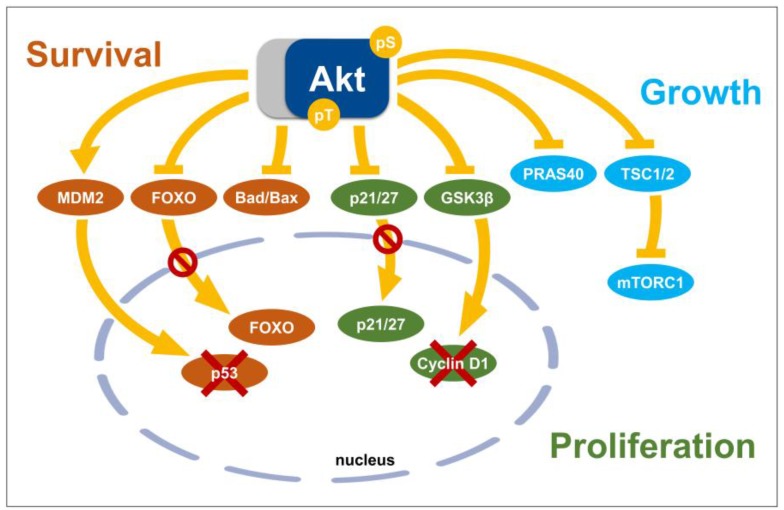
Schematic representation of Akt-substrates involved in the regulation of the cellular radiation response. Akt mediates several subcellular processes, e.g., survival, growth and phosphorylation by phosphorylating different target proteins. Phosphorylation of different Akt targets involved in survival (orange), proliferation (green) and growth (blue) leads to an activation or inhibition of their function. Arrows depict activating phosphorylation whereas blocking lines depict inhibitory phosphorylation. Nuclear translocation of p21/27 and FOXO3A is inhibited upon phosphorylation by Akt depicted by red cross.

In the following paragraphs, we will describe Akt substrates that function as direct effector proteins of Akt in processes that impact cell fate decisions upon exposure to ionizing radiation focusing on proteins involved in the regulation cell cycle progression and apoptosis (Figure 2).

#### 3.3.1. The Inhibitors of Cyclin-Dependent Kinase, p21 and p27

Cell cycle strongly changes the effects of ionizing radiation, considering that cells irradiated during the early post-mitotic G1 or pre-mitotic G2 phase are more radioresistant in comparison to more radiosensitive cells in the mitotic M, late G1 or S phases [220]. Consequently, drugs that affect cell cycle distribution impact the outcome of radiation therapy.

Cyclin-dependent kinases (CDKs) promote cell cycle progression upon binding to the respective Cyclins in a cell cycle phase dependent manner, while CDK inhibitors can inhibit their function [221]. There are two main families of CDK inhibitors: The INK4 gene family-encoded proteins p16INK4a, p15INK4b, p18INK4c, and p19INK4d bind to CDK4 and CDK6 and modulate their interaction with Cyclin D. In contrast, the members of the Cip/Kip protein family interrupt Cyclin D-, E-, A-, and B-CDK complexes [222,223]. Two members of the Cip/Kip protein family, namely p21Cip1/Waf1/Sdi1 (p21) and p27Kip1 (p27) are documented as direct Akt substrates [160,161,162,163,164,165]. Phosphorylation of p21 and p27 controls not only the affinity of the proteins for their target proteins, e.g., Cyclin-CDK complexes or the DNA synthesis regulator proliferating cell nuclear antigen (PCNA), but also the subcellular localization and the stability of these short-lived proteins with high proteasomal degradation rate [224,225,226].

Akt directly phosphorylates p21 at T145 and S146 in the carboxy-terminus close to the nuclear localization sequence. While phosphorylation of S146 significantly increased p21 protein stability and cell survival, phosphorylation at T145 inhibited binding of p21 to its substrates, e.g., PCNA or Cyclin A/E-CDK2 complexes, thereby abrogating p21-dependent inhibition of DNA replication and cell cycle progression [160,161]. Furthermore, Akt-mediated phosphorylation at T145 also triggered exclusion of p21 from the nucleus leading to its cytoplasmic accumulation. Cytoplasmic phospho-p21 was no longer able to inhibit cell cycle progression leading to unrestrained proliferation [162]. Instead phosphorylation at Ser146 led to increased p21 protein stability and improved cell survival suggesting that subcellular localization of p21 might dictate pro-survival or anti-proliferative function of p21 [160]. Of note, Akt also controls the assembly of Cyclin D1-CDK4 complexes by modulation of p21 and Cyclin D1 through the control of GSK3β-dependent degradation [160,161]. This might at least partially explain the observed activating effects on Cyclin D1-CDK4/6.

Akt also promotes phosphorylation of p27 (at T157) and this impairs nuclear translocation in human mammary epithelial cells resulting in cytoplasmic retention of p27 and cell cycle progression [165]. Moreover, Akt phosphorylation of p27 promoted its binding to 14-3-3 proteins and subsequent degradation [163].

Thus, by causing cytoplasmic retention Akt-dependent phosphorylation of p21 and p27 inactivates their suggested nuclear tumor suppressor function and this is strongly correlated with increased proliferation and poor prognosis [163,227]. However, while both, p21 and p27, have first been recognized as cell cycle inhibitors mediating growth inhibitory effects of upstream signaling pathways such as the PI3K/Akt pathway and others, they are also recognized as proteins with multifaceted functions beyond cell cycle regulation such as transcriptional regulation, cell fate determination and cytoskeletal dynamics [226]. Of note, cytoplasmic mis-localization of p27 and p21 has been associated with tumor-promoting pro-survival actions [160,228]. Though in some tumor types loss of p21 can be associated with poor prognosis, overexpression or cytoplasmic localization of p21 is another biomarker of aggressive tumors with poor prognosis in pancreatic, breast, cervical, ovarian and prostate carcinomas as well as in glioblastoma [226]. Similarly, while low amounts of nuclear p27 protein are frequently observed in diverse types of human cancer, increased cytoplasmic localization of p27 is associated with poor prognosis in specific subsets of certain tumor types, e.g., breast, cervical, ovarian, and esophageal carcinomas [229]. This suggests that particularly cytoplasmic p21 and p27 may play active roles in tumor initiation and tumor progression [226]. Interestingly, while elevated levels of p21 in aggressive tumors have been associated with increased chemoresistance [160], thus far most cell lines characterized by high radioresistance showed down-regulation of both p21 and p27 [230].

#### 3.3.2. Pro-Apoptotic Members of the Bcl-2 Family, Bad and Bax

It has been shown that phosphorylation of pro-apoptotic members of the Bcl-2 family Bad and Bax contributes to the survival-promoting effects of Akt under conditions of cell stress [231,232,233,234]. In this context, Akt-mediated phosphorylation of the BH3-only protein Bad increased binding of Bad to 14-3-3 proteins thereby abrogating its pro-apoptotic activity [235,236]. Instead, phosphorylation of the pro-apoptotic effector protein Bax was shown to preclude its translocation to mitochondria, to maintain the mitochondrial inner membrane potential, and to interrupt initiation of caspase-3-dependent intrinsic apoptosis [233]. Additional studies revealed that phosphorylation of Bax at S184 triggers its cytoplasmic translocation and promotes its heterodimerization with the anti-apoptotic Bcl-2 family members myeloid cell leukemia 1 (Mcl-1), B-cell lymphoma-extra-large (Bcl-xL), and Bcl-2-related protein A1. Phosphorylation of the pro-apoptotic Bcl-2 protein family members will lead to increased apoptosis resistance [234,236].

### 3.4. Direct Nuclear Akt Target Proteins with Roles in DSB Repair

**Figure 3:**
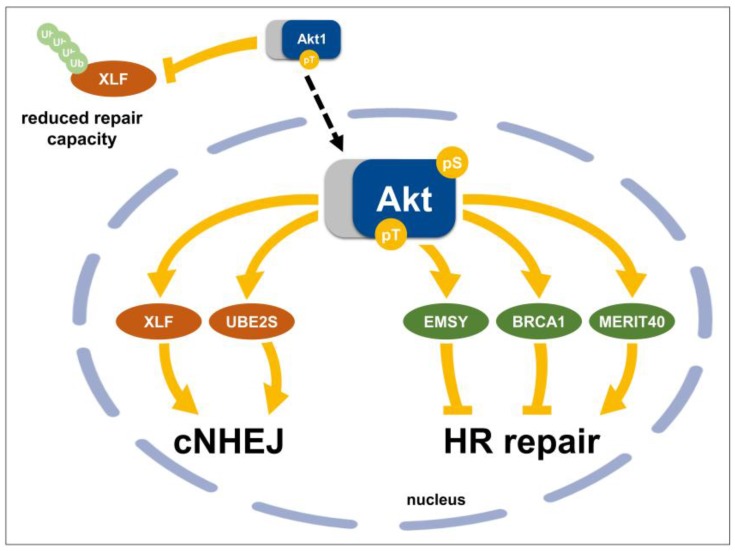
Schematic representation of the interaction of Akt with DNA repair proteins and pathways. Active Akt influences classic non-homologous end joining (cNHEJ) and homologous recombination repair (HR repair, HRR) pathways by phosphorylating proteins involved in the regulation or execution of DNA repair (cNHEJ and HRR). Proteins associated with cNHEJ are depicted in orange whereas HRR-associated proteins are labeled green. For example, upon phosphorylation XLF is removed from DNA repair complexes, excluded from the nucleus and prone to ubiquitination-dependent degradation [174] whereas phosphorylation by Akt stabilizes UBE2S thereby enhances its interaction with Ku70 and the accumulation of UBE2S-Ku70 at sites of DNA damage [171]. However, it is still unclear whether the action of Akt on XLF promotes or inhibits cNHEJ [23,174] By phosphorylating BRCA1 and MERIT40 Akt may either inhibit or promote BRCA1-dependent HRR [242,243].

Three major DNA repair pathways execute the repair of DNA DSB in mammalian cell with differences in accuracy and the dependence on cell cycle distribution: (i) classical non-homologous end joining (cNHEJ) that operates throughout the cell cycle, (ii) HRR that proceeds exclusively in G2/S phase, and (iii) alternative NHEJ (aNHEJ) that is activated in the absence of cNHEJ [64,237,238,239,240,241]. So far, Akt has been linked to DSB repair through either classical DNA-PKcs-dependent cNHEJ [23,24,42,174,171] or HRR [140,174,242,243] respectively, where it exerts either repair-promoting or inhibitory effects. For example, genetic or pharmacologic inhibition of Akt decreased DNA-PKcs-dependent DSB repair and increased the cytotoxicity of chemotherapy and ionizing radiation in preclinical investigations [42,60,244,245,246,247]. Moreover, loss of Akt1 and/or Akt2 abrogated the ability of colorectal DLD-1 and HCT116 cell lines to undergo DNA DSB repair upon ionizing radiation [248]. Instead, overexpression of Akt3 in a genetic model of PDGF-driven murine glioma [8] or of the clinically relevant activating mutation Akt1-E17K in murine prostate cancer cells facilitated the repair of radiation-induced DNA damage, thereby increasing radiation resistance [23]. But, Akt can also exert inhibitory actions on the proper repair of DNA damage [243,141,249]. In the following paragraphs we highlight current knowledge about important nuclear proteins with suspected regulation by Akt and their positive or negative effect on DNA DSB repair (Figure 3).

#### 3.4.1. Chk1 (Checkpoint Kinase 1)

Chk1 is involved in the regulation of the G2/M transition and induces G2 arrest upon DNA damage. Through this process, Chk1 inhibits proliferation and avoids the propagation of DNA lesions [250,251]. However, active Akt mediates inhibitory phosphorylation of Chk1 at S280 thereby allowing for cell cycle progression and survival of cells with unrepaired DNA damage [141]. Instead, depletion of Akt in BRCA1-deficient cells enabled Chk1-Rad51 interaction with subsequent recruitment to DNA damage sites and enhanced HRR [140]. Interestingly, the failure of G2 cells to activate Chk1 and to avoid mitotic entry upon IR can be abolished by pharmacological inhibition of Akt [142]. Taken together, Akt inhibition seems to be required to achieve Chk1-mediated G2 arrest and avoid mitosis in cells with damaged DNA.

#### 3.4.2. BRCA1 (Breast Cancer 1)

The HRR protein BRCA1 is frequently mutated in cancer and was described as a biomarker in breast and ovarian cancer [252,253]. BRCA1 forms diverse complexes, e.g., BRCA1-A, BRCA1-B, BRCA1-C, and the BRCA1/PALB2/BRCA2 complex, and plays diverse roles in the regulation of checkpoint activation, repair pathway choice, and HRR efficiency in a context-dependent manner [254,255]. Interestingly, BRCA1 is a direct Akt substrate that can be phosphorylated at two sites—S694 and T509 [138,139]. However, the role of Akt in BRCA1-phosphorylation and BRCA1-dependent DNA repair is still not well understood. Plo and colleagues observed a correlation between constitutively active Akt1 and impairment of HRR by inhibiting BRCA1-foci formation [243]. The authors attributed the inhibitory effects of Akt to enhanced cytoplasmic retention of BRCA1 and Rad51 as well as to impaired formation of nuclear BRCA1-foci upon IR [243]. Rather, expression of both, myr-Akt and BRCA1, significantly improved survival of irradiated MCF7 cells in comparison to MCF7 cells transfected only with BRCA1 or myr-Akt alone [139]. Improved survival of myr-Akt/BRCA1-transfected MCF-7 cells was associated with enhanced nuclear BRCA1-accumulation suggesting that active Akt may be important to nuclear assembly of BRCA1 complexes and modulation of HRR [139]. Thus, the role of Akt-mediated BRCA1 phosphorylation for DNA repair remains controversial and more studies are needed to gain a better understanding of the interaction between Akt and BRCA1 and its influence on DNA repair and the fate of irradiated cancer cells.

#### 3.4.3. MERIT40 (Mediator of Rap80 Interactions and Targeting 40 kDa)

As mentioned above, BRCA1 cooperates and forms complexes with various other proteins to promote HRR. One of these complexes, BRCA1-A, seems to be critical for the accumulation of BRCA1 at sites of DNA damage. BRCA1-A involves the ubiquitin-binding motif (UIM)-containing protein RAP80, the coiled-coil domain protein CCDC98/Abraxas, the deubiquitinating enzyme BRCC36, as well as BRCC45, BARD1, and MERIT40 [252,254,256]. MERIT40 positively influences HRR by enhancing assembly and stability of BRCA1 complexes at sites of DNA damage ends [256,257]. Moreover, MERIT40 cooperates with BRCA2 in resolving interstrand cross-links [255]. Herein, MERIT40 has been identified as substrate of Akt; Akt-mediated MERIT40 phosphorylation at S29 facilitated assembly of BRCA1 complexes in response to doxorubicin-induced DNA damage, execution of HRR and survival of doxorubicin-treated breast cancer cell lines (MCF7, SUM159 and T47D) independently of the Akt isoform and of mTORC1 [242]. Of note, genetic inhibition of Akt by shRNA led to inhibition of MERIT40 phosphorylation and an impaired DNA damage response [242]. However, the role of Akt-mediated phosphorylation of MERIT40 in the cellular response and the repair of radiation-induced DNA DSB has not yet been investigated and therefore remains elusive. It appears that phosphorylation of MERIT40 by active Akt may also facilitate HRR in irradiated cancer cells, comparable to its role in the response to doxorubicin.

#### 3.4.4. EMSY (BRCA2-Interacting Transcriptional Repressor)

Interestingly, EMSY constitutes another Akt substrate with potential impact on DNA repair if overexpressed [249]. Breast cancer and ovarian cancer cells show strong amplification of EMSY and this is associated with poor prognosis [258]. EMSY can bind to BRCA2, a protein implicated in transcriptional regulation and DNA repair, leading to deactivation and transcriptional repression of BRCA2 [144,145,249,259]. While overexpression of EMSY repressed interferon-stimulated genes in breast and ovarian cancer cells in a BRCA2-dependent manner, its knockdown had opposite effects [144,145]. In this context, Akt1-dependent phosphorylation of EMSY at S209 seems to be important to the interferon response [144,145], but not for decreasing HRR efficacy. Instead, Akt1-dependent phosphorylation of EMSY at T207 might be critical for reducing Rad51 foci formation and impairment of HRR [144,145]. Because of a very recent discovery of EMSY as an Akt1-substrate, the role of Akt-mediated phosphorylation of EMSY for its influence on HRR requires further definition.

#### 3.4.5. XLF (Cernunnos/XRCC4-Like Factor)

XLF belongs to the cNHEJ proteins, but its specific function remains elusive. XLF interacts with the XRCC4-Ligase IV complex during cNHEJ in vitro and in vivo [260,261]. Herein, the interaction between XLF and XRCC4 is necessary to create filaments and bridge broken DNA ends thereby facilitating end-ligation by DNA ligase IV [174,262,263]. Of note, RNAi mediated inhibition of XLF impaired DNA repair and enhanced radiosensitivity of U2OS cells [260].

While in vitro experiments suggested that DNA-PKcs is able to phosphorylate XLF at two sites—S245 and S251—in vivo experiments revealed that DNA-PKcs phosphorylates only S245, whereas S251 requires ATM-activation to be phosphorylated [264]. But phosphorylation of these sites did not significantly affect the repair of radiation-induced DSB suggesting that pS245 and pS251 might not play a critical role for DSB repair [264]. Interestingly, Liu and colleagues recently identified XLF as Akt1-target protein [174]. The authors revealed that Akt1-dependent XLF phosphorylation promotes the binding of XLF to 14-3-3β proteins evoking its nuclear exclusion, dissociation of cNHEJ complex and impaired DNA repair via cNHEJ [174]. Surprisingly, neither Akt2 nor Akt3 were able to phosphorylate XLF [174]. In contrast, our own data revealed that overexpression of the activation-associated Akt1 mutants Akt1-E17K and Akt1-T308D/S473D accelerated DNA repair and promoted radiation resistance in murine prostate cancer cells and murine embryonic fibroblasts [23]. Since neither phosphorylation of XLF by DNA-PKcs nor by ATM seems to be essential for DNA repair, we proposed that Akt1-mediated phosphorylation of XLF may increase DNA repair dynamics by accelerating dissociation of cNHEJ complexes after completed DNA repair [23].

#### 3.4.6. UBE2S (Ubiquitin-Conjugating Enzyme E2S)

Overexpression of ubiquitin-conjugating enzyme E2S (UBE2S) in malignant tumors has been linked to tumor growth, invasion and metastasis [265]. Surprisingly, Hu and colleagues recently proposed UBE2S as a novel Akt1-substrate with a function in DSB-repair via cNHEJ: Akt1 physically interacted with and phosphorylated UBE2S at T152 thereby enhancing its stability and association with Ku70 [171]. Importantly, knockdown of UBE2S led to sensitization of glioblastoma cells to genotoxic chemotherapy with Etoposide. Mechanistically, Akt1-mediated phosphorylation of UBE2S led to enhanced Ku70-UBE2S binding, accumulation of UBE2S-Ku70 at sites of DNA damage and improved DNA DSB repair via cNHEJ. Though initial data including our own unpublished work suggest a function of the Akt1-UBE2S axis in the repair of radiation-induced DNA DSB, further experimental work is required to substantiate conditions, mechanisms and consequences of the Akt/UBE2S interaction, if we aim to exploit this interaction for therapeutic intervention [23,171,172].

##### Perspective

Akt plays an active role in promoting DNA DSB repair through DNA-PKcs-dependent NHEJ or HRR. Herein, aberrant activation of Akt mostly provides cancer cells with increased ability to repair DNA DSB and this might help the cancer cells to escape from genotoxic chemotherapy or radiotherapy. The repair-promoting activity makes aberrantly activated PI3K/Akt signaling a promising target for radio-/chemosensitization. The identification of Akt substrates with impact on cNHEJ or HRR and of the mechanisms underlying their repair-modulating actions will help us to define biomarkers for altered repair in cancer cells and to exploit the therapeutic potential of Akt-targeting strategies in combination treatments in the future.

## 4. Conclusions and Outlook

PI3K/Akt pathway is one of the most frequently dysregulated signaling pathways in human cancer and impacts on almost all aspects of malignant cancer growth, the so-called “hallmarks of cancer”. This review describes the multifaceted regulatory aspects of this complex signaling network with a focus on localized activation/deactivation cycles and compartment-specific Akt target proteins. 

Moreover, this review highlights of the involvement of diverse Akt substrates in subcellular processes with importance to the DNA damage response, DNA repair, and the maintenance of genomic stability that are expected to impact tumor initiation and progression. The finding that aberrant activation of the survival kinase Akt mostly enhances the repair of DNA DSB through either classical DNA-PKcs-dependent cNHEJ or HRR suggests that aberrant activation of Akt may help the cancer cells to increase the overall capacity for DNA repair upon exposure to genotoxic therapies and thus promotes resistance to genotoxic therapies. A common feature of active Akt in the nucleus seems to be its regulatory effect on the formation of protein-protein/multiprotein repair complexes and the dynamics of complex-dissociation. We assume that through this Akt might facilitate or even accelerate the formation and resolution of DNA repair foci ensuring survival under therapy.

Some of the described Akt substrates have only recently been identified and are currently explored in preclinical investigation suggesting novel opportunities for therapeutic intervention in the future. Herein promising candidates are nuclear Akt target proteins with a documented role in the repair of DNA DSB. Targeting miRNAs involved in regulation of the PI3K/Akt pathway may be another innovative approach to overcome radioresistance of cancer cells caused by PI3K/Akt pathway alterations. Deregulated expression of miRNAs regulating the PI3K/Akt pathway may not only be used as innovative therapeutic targets but also as biomarkers for patient selection in clinical trials. 

A comprehensive understanding of the complex PI3K/Akt signaling network, its localized activation/deactivation cycles and its compartment-specific target proteins will help to exploit the full potential of pathway inhibitors in cancer therapy including combination with genotoxic chemotherapy and radiotherapy. The role of PI3K/Akt signaling in the cellular radiation response is a field of intense research in radiation biology with a high potential for groundbreaking findings in the future.

## Figures and Tables

**Table 1:** Regulation of the PI3K/Akt pathway with miRNAs.

miRNA Name	Function	Reference
**miR-20a**	Activation of mir-20a correlated with decreased activation of PTEN leading to higher radioresistance. Inhibition of miR-20a by anti-miR-20a led to cell radiosensitization. PI3K inhibitor LY294002 could radiosensitize hepatocellular carcinoma cells.	[110]
**miR-21** **miR-95**	Upregulation of miR-21 and miR-95 expression in lung cancer cells correlating with poor prognosis. Radiosensitization by applying anti-miR-21 and anti-miR-95.	[116]
**miR-22**	miR-22 activates PI3K/Akt pathway inducing enhanced proliferation of chronic lymphocytic leukemia (CLL) B-cells.	[117]
**miR-31**	Inhibiting effects of miR-31 evoked downregulation of PI3K/Akt pathway in lung adenocarcinoma cells.	[115]
**miR-101-2** **miR-125b-2 miR-451a**	Exogenous expression of miR-101-2, miR-125b-2, and miR-451a suppressed tumor growth in gastric cancer through decreasing the expression of PI3K/Akt pathway.	[118]
**miR-126**	miR-126 suppressed proliferation of undifferentiated thyroid carcinoma through repressing PI3K/Akt pathway.	[111]
**miR-203**	miR-203 is critical factor in radioresistance of nasopharyngeal carcinoma cells by targeting IL8/AKT signaling. This effect could be abolished by an agomir. Overexpression of miR-203 could achieve radiosensitization by affecting DNA repair as well as the PI3K/Akt pathway in malignant glioma cells.	[112]
**miR-205**	Enhanced expression of miR-205 evokes downregulation of PTEN resulting in enhanced Akt phosphorylation and radioresistance.	[119]
**miR-302**	Downregulation of miR-302 evokes elevated level of phosphorylated Akt. Restoration of miR-302 expression returned this effect and sensitized cells to radiotherapy.	[113]
**miR-519a**	miR-519a can promote tumor growth in hepatocellular carcinoma targeting PTEN/PI3K/Akt pathway.	[120]

**Table 2:** Documented Akt target proteins.

Target Name	Function	References
**BAD**	Pro-apoptotic protein. Phosphorylation by Akt inhibits its function and promotes cell survival.	[136,137]
**BRCA1**	Breast cancer susceptibility gene product and tumor suppressor. Phosphorylation by Akt alters its function, perhaps by preventing nuclear localization.	[138,139]
**CHK1**	DNA damage effector that regulates G2/M transition during DNA damage. Phosphorylation by Akt inhibits its function by preventing phosphorylation by ATM/ATR.	[140,141,142]
**Cot**	Oncogene. Phosphorylation by Akt induces NF-kB-dependent transcription.	[143]
**EMSY**	Oncogenic interacting partner of BRCA2. EMSY overexpression disrupts the BRCA2/RAD51 interaction.	[144,145]
**FANCA**	ATPase involved in DNA repair. Phosphorylation by Akt negatively regulates its function.	[146,147]
**FOXO1A**	Transcription factor involved in cell cycle arrest, apoptosis, and glucose metabolism. Phosphorylation by Akt causes export from the nucleus and inhibits its activity.	[148,149,150]
**FOXO3A**	Transcription factor involved in cell cycle arrest and apoptosis. Phosphorylation by Akt causes export from the nucleus and inhibits its activity.	[151,152,153]
**FOXO4**	Transcription factor involved in cell cycle arrest, apoptosis, and insulin signaling. Phosphorylation by Akt causes export from the nucleus and inhibits its activity.	[154]
**Lamin A/C**	Plays a role in nuclear assembly, chromatin organization, nuclear membrane and telomere dynamics. Phosphorylation by Akt promotes its degradation.	[155,156]
**Mdm2**	As an E3 ubiquitin-protein ligase Mdm2 mediates ubiquitination of p53/TP53.	[124,157,158]
**Merit40**	Component of the BRCA1-A complex that contributes to DNA repair. Phosphorylation by Akt leads to enhanced DNA repair and survival.	[159]
**p21**	p21 as a CDK-inhibitor regulates cell cycle and survival. Phosphorylation by Akt leads to release from PCNA that results in elevated progression.	[160,161,162]
**p27**	Cyclin-dependent kinase inhibitor that mediates G1 arrest. Phosphorylation by Akt promotes 14-3-3 binding and cytoplasmic localization resulting in enhanced proliferative effect.	[163,164,165]
**PRAS40**	Binds to and inhibits mTOR. Phosphorylation causes 14-3-3 binding and facilitates its phosphorylation by mTORC1.	[166,167]
**PRP19**	PRP19is a member of the spliceosome that also functions in DNA double strand break repair. Phosphorylation by Akt allows 14-3-3 binding and promotes its degradation.	[168]
**TSC2**	Tumor suppressor that can inhibit mTOR. Phosphorylation by Akt inhibits its function.	[169,170]
**UBE2S**	Ubiquitin-conjugating enzyme E2S plays a role in NHEJ complex. Phosphorylation by Akt enhances its stability by inhibiting proteasomal degradation.	[171,172]
**Wee1**	Inhibits cell cycle progression. Phosphorylation by Akt inhibits its function associated with changed localization of Wee1 from nuclear to cytoplasmic.	[173]
**XLF**	Involved in NHEJ and promotes the Ligase IV recruitment to the DNA damage site. XLF interacts with XRCC4 to create long filaments promoting ligation of DNA broken ends. Phosphorylation by Akt negatively affects DNA repair.	[174]
